# Drug‐induced interstitial lung disease associated with dasatinib coinciding with active tuberculosis

**DOI:** 10.1002/rcr2.654

**Published:** 2020-08-27

**Authors:** Nozomi Tani, Yusuke Kunimatsu, Izumi Sato, Yuri Ogura, Kazuki Hirose, Takayuki Takeda

**Affiliations:** ^1^ Department of Respiratory Medicine Japanese Red Cross Kyoto Daini Hospital Kyoto Japan

**Keywords:** Chronic myeloid leukaemia, dasatinib, drug‐induced interstitial lung disease, tuberculosis, tyrosine kinase inhibitor

## Abstract

A 69‐year‐old woman was diagnosed with a breakpoint cluster region‐Abelson‐positive chronic myeloid leukaemia and treated with dasatinib for 14 months. She presented with one month of high‐grade fever and persistent dry cough. Chest computed tomography revealed non‐segmental subpleural consolidation, ground‐glass opacities, and interlobular septal thickening. The bronchoalveolar lavage (BAL) and transbronchial lung biopsy confirmed a diagnosis of drug‐induced interstitial lung disease (ILD) associated with dasatinib. Then, systemic corticosteroid treatment was initiated, which was effective and the interstitial shadow disappeared after two weeks. The acid‐fast bacilli culture test of BAL fluid after three weeks was positive for *Mycobacterium tuberculosis*, and combination therapy with four antituberculosis drugs was added. It is known that drug‐induced ILD and susceptibility to infection associated with dasatinib occur in a dose‐dependent manner. This is the first case of dasatinib‐induced ILD which coincided with active tuberculosis.

## Graphical abstract

Drug‐induced interstitial lung disease (ILD) and immunosuppression are known to develop in a dose‐dependent manner, and we herein describe the first case of dasatinib‐induced ILD which coincided with active tuberculosis.

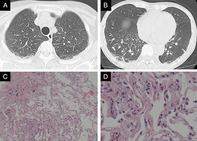

## Introduction

Breakpoint cluster region‐Abelson (BCR‐ABL) tyrosine kinase inhibitor (TKI), represented by imatinib, has revolutionized the treatment strategy for chronic myeloid leukaemia (CML). Dasatinib is the second‐generation TKI, whose common adverse events (AEs) are pleural effusion and drug‐induced interstitial lung disease (ILD). Dasatinib also suppresses cell‐mediated immunity, which makes relevant patients susceptible to microorganisms including bacterium, viruses, and tuberculosis. Drug‐induced ILD and immunosuppression are known to develop in a dose‐dependent manner, and we herein describe the first case of dasatinib‐induced ILD which coincided with active tuberculosis.

## Case Report

**Figure 1:**
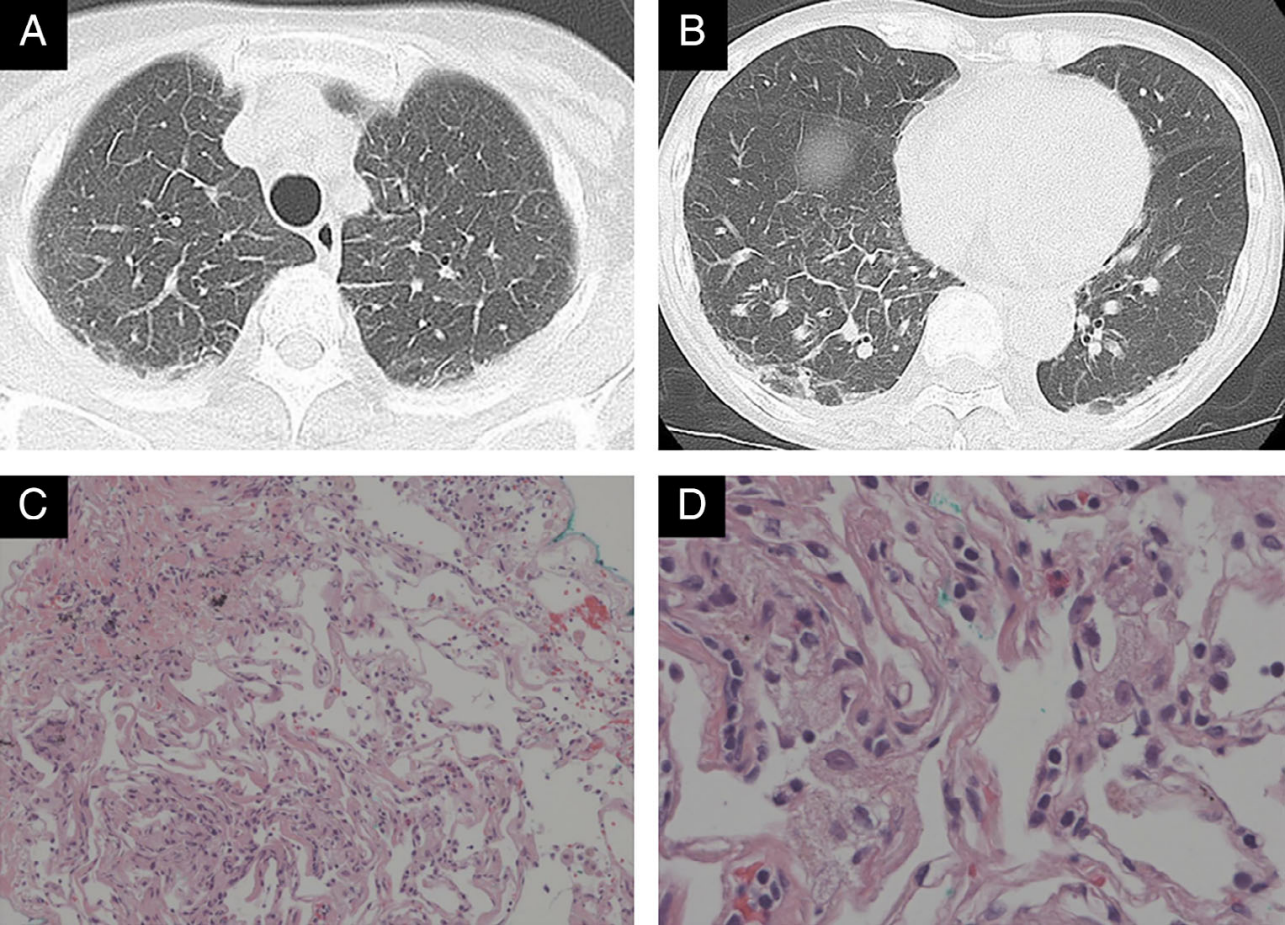
Chest computed tomography (CT) findings on admission. Chest CT demonstrated non‐segmental subpleural consolidation, ground‐glass opacities, forming reversed halo sign in some areas, and interlobular septal thickening, without pleural effusion (A, B). Subpleural consolidation and interlobular septal thickening were predominant in bilateral lower lobes (B) over upper lobes (A). Pathological findings of transbronchial lung biopsy were non‐specific with lymphocyte infiltration into interlobular septum (C, D), without caseous necrosis which is specific to tuberculosis. Magnification power was 100× and 400× in (C) and (D), respectively.

**Figure 2:**
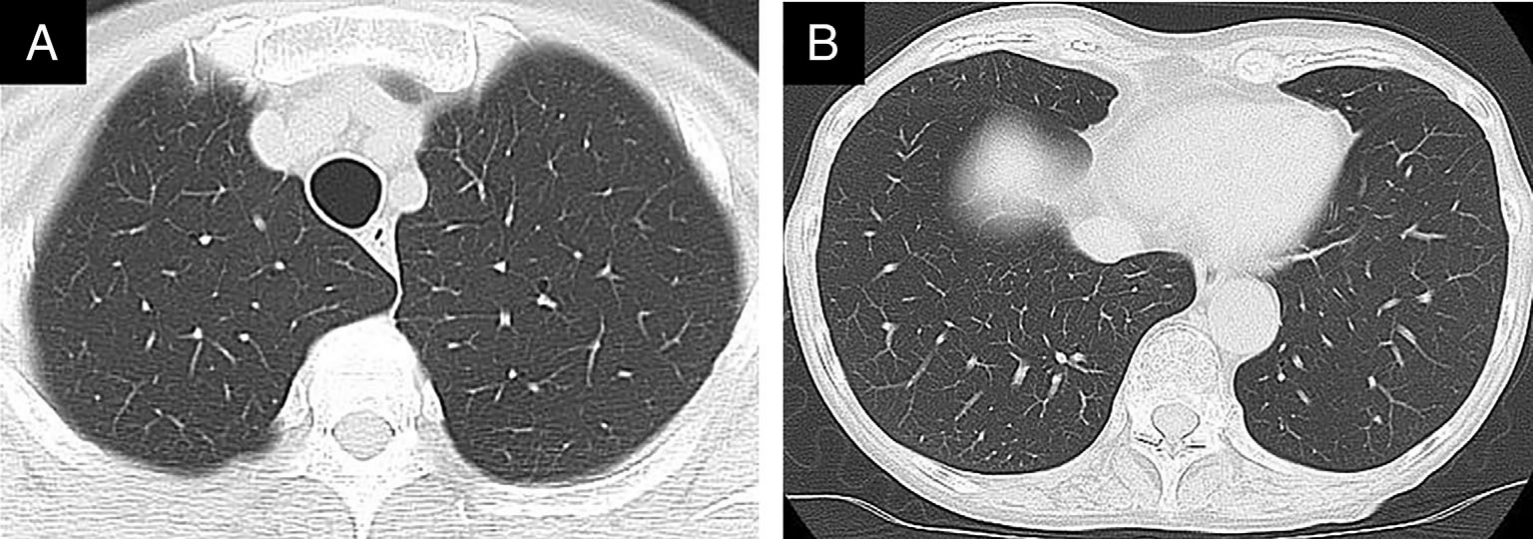
Chest computed tomography (CT) findings two weeks after corticosteroid treatment. Chest CT obtained two weeks after corticosteroid treatment showed substantial improvement in the interstitial shadows both in bilateral upper (A) and lower (B) lobes.

A 69‐year‐old woman who had been diagnosed with BCR‐ABL‐positive CML developed a lymphoblastic blast crisis. She received remission induction therapy with dasatinib at a dose of 70 mg twice daily and prednisolone (PSL) at a dose of 85 mg per day. After four cycles of intensive consolidation therapy (dasatinib, 100 mg once daily; PSL, 60 mg; cyclophosphamide, 1200 mg on day 1; daunorubicin, 40 mg on days 1–3; vincristine, 1.7 mg on days 1, 8, 15, and 22), bone marrow biopsy revealed a complete remission, which was followed by a maintenance therapy with dasatinib at a dose of 100 mg once daily. Dasatinib was continued for 14 months, when she complained of high‐grade fever reaching 39°C, dry cough, and dyspnoea on exertion. The symptoms did not improve after one month of treatment with antibiotics, antitussive agents, and antipyretics, then she was referred to our department. SpO_2_ (peripheral capillary oxygen saturation) was 97% under oxygen administration at 2 L/min. The laboratory findings on admission were as follows: white blood cell count, 9500/μL with 34% of neutrophils (Neut.), 56% of lymphocytes (Lymph.), and 3% of eosinophil (Eos.); lactate dehydrogenase, 296 U/L; Krebs von den Lungen‐6 (KL‐6), 193 U/mL; C‐reactive protein, 4.25 mg/dL; autoantibodies, negative; tuberculosis‐specific interferon γ, negative; and serological markers of fungal infections including β‐d‐glucan, negative. Chest computed tomography (CT) revealed non‐segmental subpleural consolidation, ground‐glass opacities, and interlobular septal thickening, without pleural effusion (Fig. 1A, B). The result of bronchoalveolar lavage (BAL) which was performed from right B^5^, where there was no abnormal shadow other than interlobular septal thickening, showed the increased number of cells with lymphocyte predominance (total cell counts, 8.7 × 10^5^/mL; Neut., 0%; Lymph., 76%; and Eos., 0%). Pathological findings of transbronchial lung biopsy, which was performed from right B^9^b, B^4^a, and B^2^b, were non‐specific with lymphocyte infiltration into interlobular septum (Fig. 1C, D), without intra‐alveolar polypoid lesion which is specific to organizing pneumonia patten, or caseous necrosis which is specific to tuberculosis. Dasatinib was the most probable drug with a total score of 6 (probable) using the drug interaction probability scale [1]. Since dasatinib was indispensable in the maintenance of CML remission, dasatinib was continued without dose reduction. Under the diagnosis of drug‐induced ILD associated with dasatinib, PSL was initiated at a dose of 20 mg (0.5 mg/kg). Cough and high‐grade fever disappeared soon after the treatment. Subpleural consolidation, ground‐glass opacities, and interlobular septal thickening disappeared on CT after two weeks (Fig. 2). During this period, dasatinib was administered at the same dosage without reduction. Although acid‐fast bacilli stain and *Mycobacterium tuberculosis* polymerase chain reaction (PCR) were both negative in the BAL fluid, the acid‐fast bacillus culture turned out to be positive for *M. tuberculosis* after three weeks. Combination antituberculosis therapy was immediately added with rifampicin, isoniazid, ethambutol, and pyrazinamide. An intensive phase of two months was followed by a continuation phase with rifampicin and isoniazid for seven months. The treatment period was extended due to impaired cell‐mediated immunity under BCR‐ABL‐positive CML, continuation of dasatinib, and PSL treatment. PSL was gradually tapered to a dose of 10 mg/day without relapse of dasatinib‐induced ILD.

## Discussion

Dasatinib has been increasingly used in the treatment of CML. Drug‐induced ILD associated with dasatinib exhibiting ground‐glass opacities and interlobular septal thickening has been reported in two case series [2, 3], while pleural effusion being the most common AE. Dasatinib‐induced ILD occurs in a dose‐dependent manner, as the ILD improved after interruption of dasatinib, and was well controlled with a dose reduction in 75% of such cases [2]. This perspective was supported by the results of the phase 3 trial comparing the groups receiving 70 mg of dasatinib twice daily and 100 mg once daily for imatinib‐resistant CML, in which pleural effusion was observed in a dose‐dependent manner [4]. In addition, the exudative pleural effusion and dasatinib‐induced ILD are caused by immune response which is irrelevant to the dasatinib‐specific fluid retention mechanism, because pleural effusion and BAL fluid analysis showed lymphocyte predominance and ILD was reversible. Dasatinib inhibits not only BCR‐ABL tyrosine kinase, but also Src family kinase. Inhibition of Src family kinase is known to cause autoimmune diseases such as systemic lupus erythematosus. Although no autoimmune AEs associated with dasatinib were reported in clinical trials, these AEs could happen due to the inhibitory effects of dasatinib on Src family kinases or other pathways in the immune system. On the other hand, it has also been reported that dasatinib suppresses activation and proliferation of CD8‐positive T cells in a dose‐dependent manner [5]. *Mycobacterium tuberculosis* is phagocytosed by macrophages and dendritic cells, and antigens derived from it migrate to the hilar lymph nodes, inducing immune responses by CD8‐positive T cells. As CD8‐positive T cells destroy tuberculosis‐infected cells, impaired cell‐mediated immunity by dasatinib increases the risk of tuberculosis. Active tuberculosis during imatinib treatment was reported in which both acid‐fast bacilli stain and *M. tuberculosis* PCR were negative, with acid‐fast bacillus culture positive for tuberculosis [6]. Thus, tuberculosis cannot be ruled out even in cases without specific findings of tuberculosis until confirming negative acid‐fast bacillus culture of appropriate specimen. As patients receiving BCR‐ABL TKIs often develop atypical infection due to impaired cell‐mediated immunity, opportunistic infections including tuberculosis should be fully considered during the treatment. To the best of our knowledge, this is the first case of developing dasatinib‐induced ILD coinciding with active tuberculosis. As both drug‐induced ILD and opportunistic infections develop in a dose‐dependent manner, coincidence of them should be considered. Antituberculosis treatment period should be extended in cases under impaired cell‐mediated immunity.

### Disclosure Statement

Appropriate written informed consent was obtained for publication of this case report and accompanying images.

## References

[1] Horn JR , Hansten PD , and Chan LN . 2007 Proposal for a new tool to evaluate drug interaction cases. Ann. Pharmacother. 41:674–680. 17389673 10.1345/aph.1H423

[2] Bergeron A , Réa D , Levy V , et al. 2007 Lung abnormalities after dasatinib treatment for chronic myeloid leukemia. Am. J. Respir. Crit. Care Med. 176:814–818. 17600277 10.1164/rccm.200705-715CR

[3] Ito Y , Tanigawa M , Iwamoto K , et al. 2019 Interstitial pneumonitis associated with dasatinib: two case reports and literature review. Respir. Investig. 57:506–509. 10.1016/j.resinv.2019.06.002 31311724

[4] Shah NP , Kim DW , Kantarjian HM , et al. 2007 Dasatinib 50 mg or 70 mg BID compared to 100 mg or 140 mg QD in patients with CML in chronic phase (CP) who are resistant or intolerant to imatinib: one‐year results of CA180034. J. Clin. Oncol. 25:A7004.

[5] Fei F , Yu Y , Schmitt A , et al. 2008 Dasatinib exerts an immunosuppressive effect on CD8+ T cells specific for viral and leukemia antigens. Exp. Hematol. 36:1297–1308. 18619726 10.1016/j.exphem.2008.05.002

[6] Daniels JMA , Vonk‐Noordegraaf A , Janssen JJWM , et al. 2009 Tuberculosis complicating imatinib treatment for chronic myeloid leukemia. Eur. Respir. J. 33:670–672. 19251803 10.1183/09031936.00025408

